# 

**Published:** 1894-09-01

**Authors:** 


					?HE HOSPITAL. Supplied under Royal Warrant to Her Majesty the Queen.
Sfipr. lsr, 1894.
" THE king OF NATURAL TABLE WATERS. ttT 1 ? ??
dopMiu> t h s uo^annia
till
OR MICH HOSPl TAL
No. 414. Vol. XVI. WITH EXTRA NURSING SUPPLEMENT. ^ice twopence.
Saturday, Sept. 1st, 1894. '?r K^A^siia.','^S

				

